# Class II Correction and Crowding Treatment Using In-House Direct Printed Clear Aligners: A Literature Review and Case Report

**DOI:** 10.7759/cureus.65024

**Published:** 2024-07-21

**Authors:** Hoang Viet, Tran Hung Lam, Nguyen Ngoc Phuc, Nguyen Ngoc Lenh, Dang Thi Nhu Thao

**Affiliations:** 1 Orthodontics, Sai Gon Dental Hospital, Ho Chi Minh, VNM; 2 Prosthodontics, Van Lang University, Ho Chi Minh, VNM; 3 Periodontology and Implantology, Van Lang University, Ho Chi Minh, VNM; 4 Orthodontics, Private Laboratory, Sai Gon Dental Hospital, Ho Chi Minh, VNM; 5 Orthodontics and Pedodontics, Van Lang University, Ho Chi Minh, VNM

**Keywords:** direct printed clear aligner, orthodontic crowding, class ii malocclusion, digital orthodontics, clear aligner

## Abstract

Clear aligner therapy has significantly improved orthodontic treatment by offering patients a more aesthetically pleasing option compared to traditional braces. This literature review and case report specifically focus on the effectiveness of directly printed clear aligners in treating Class II malocclusions and crowding. Class II malocclusions are characterized by excessive overjet, which often results from skeletal or dental discrepancies between the upper and lower jaws. Crowding refers to the lack of space for teeth within the dental arch, leading to misalignment and potential functional issues.

The review and case report highlight the increasing importance of directly printed clear aligners in modern orthodontics and provide clinicians with a valuable tool to effectively address complex malocclusions and crowding while also meeting patient needs for discretion and comfort. Further research is necessary to validate the long-term stability and outcomes of directly printed clear aligner therapy in various orthodontic cases. A detailed case report demonstrates the successful treatment of a patient with Class II malocclusion and mild crowding using directly printed clear aligners. Treatment outcomes include improvements in dental alignment, occlusion, and facial aesthetics, showcasing the effectiveness of this innovative approach.

## Introduction

In contemporary orthodontics, the management of Class II malocclusion and dental crowding has evolved significantly with the advent of clear aligner therapy [1,2]. Class II malocclusion is characterized by a discrepancy between the maxilla and mandible, often resulting in an anterior protrusion of the upper teeth relative to the lower teeth. Traditionally, treatment has involved fixed appliances, such as braces, and functional appliances like headgear. However, clear aligner therapy has gained popularity due to its aesthetic appeal and patient convenience. Clear aligners utilize a series of removable, transparent trays that gradually move teeth into their desired positions. They are custom-designed based on digital impressions or scans of the patient's teeth. Direct printed clear aligners represent a technological advancement where aligners are produced directly from digital models, bypassing the need for physical models and improving treatment efficiency [3]. Digital applications in dentistry and orthodontics always help the clinician have better control of the treatment and decrease the time chairside [4-6]. This literature review and case report aim to explore the efficacy and treatment outcomes of using direct printed clear aligners in correcting Class II malocclusion and addressing dental crowding.

## Case presentation

**Figure 1:**
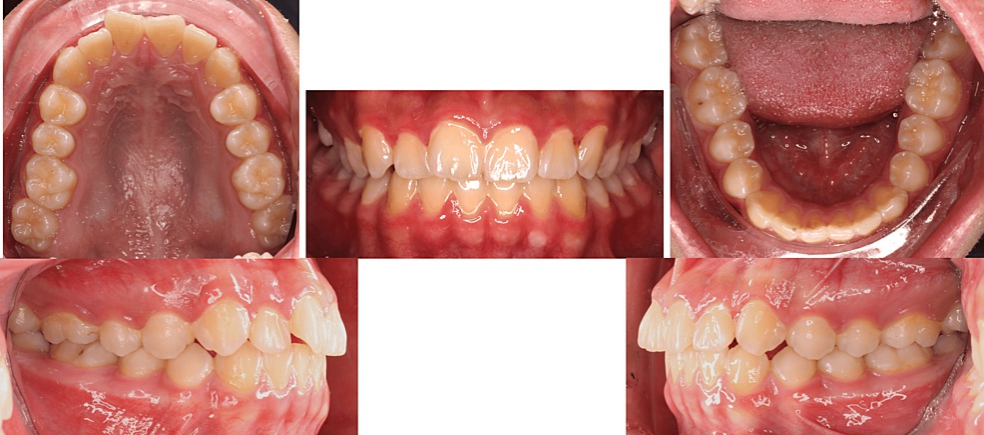
Intraoral pictures pre-treatment

**Figure 2:**
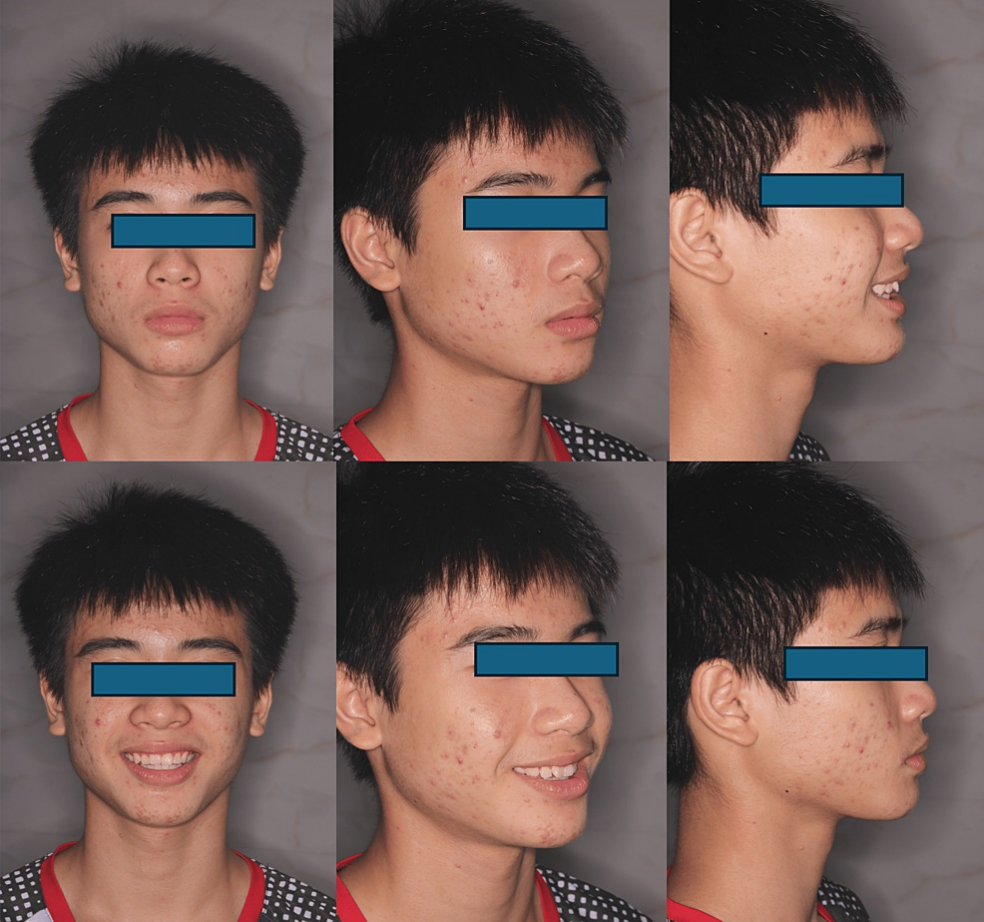
Extraoral pictures pre-treatment

**Figure 3:**
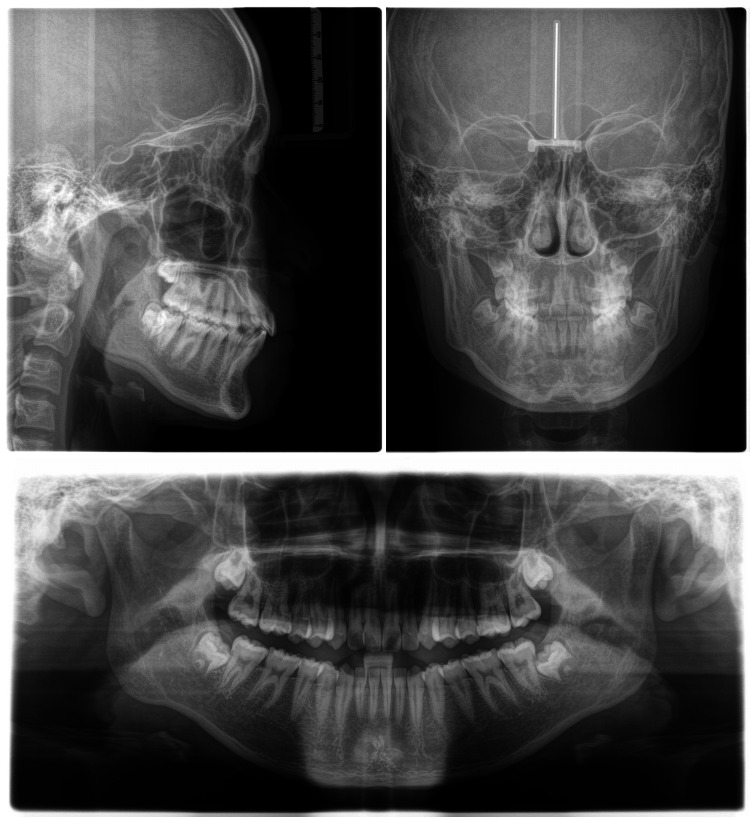
X-rays pre-treatment

**Figure 4:**
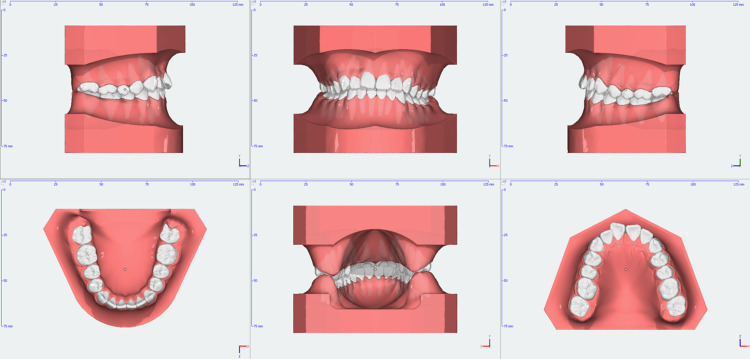
Pre-treatment 3D models

The 14-year-old male patient came in with the main concern of overcrowding and protrusion of his upper and lower front teeth. Upon examination, it was found that the patient had a straight profile with a normal appearance of the cheeks when closing the mouth (no TMJ symptoms), a Class I molar relationship, a Class II canine relationship, overbite of 2 mm, overjet of 4 mm, normal upper and lower arch form. Specifically, in the upper arch, there was 3 mm of crowding and protrusion in the anterior teeth, and in the lower arch, there was 1 mm of crowding with a normal curve of Spee. The midline of the upper and lower is coincident with the facial midline. In terms of the cephalometric analysis, the patient was a skeletal class II with ANB 4.3°, protruded position of the maxillary and normal position of the mandible (SNA 87.1°, SNB 82.8°), normal lower facial height (FMA 23.4°), proclination of the upper and lower incisors (U1-SN 117.3°, L1-MP 96.2°). The pretreatment records of the patient are shown in Figures 1-4.

Treatment objectives

The objectives of the treatment were to eliminate the anterior crowding and class II malocclusion, achieve satisfactory smile aesthetics and masticatory function, and achieve stable occlusion and treatment outcome in the long term.

Potential alternative treatments

In discussions with the patient about this case, we presented several treatment options, including the choice between the current treatment plan and traditional braces. However, the patient’s parents prefer the aesthetic option for their son, so they have decided on non-extraction treatment, sequence distalization [7] of the upper to correct class II malocclusion, alignment of upper, lower and upper incisors retraction using direct printed in-house clear aligners. In this case, both braces and clear aligners were suitable options for the patient.

Treatment progress

**Figure 5:**
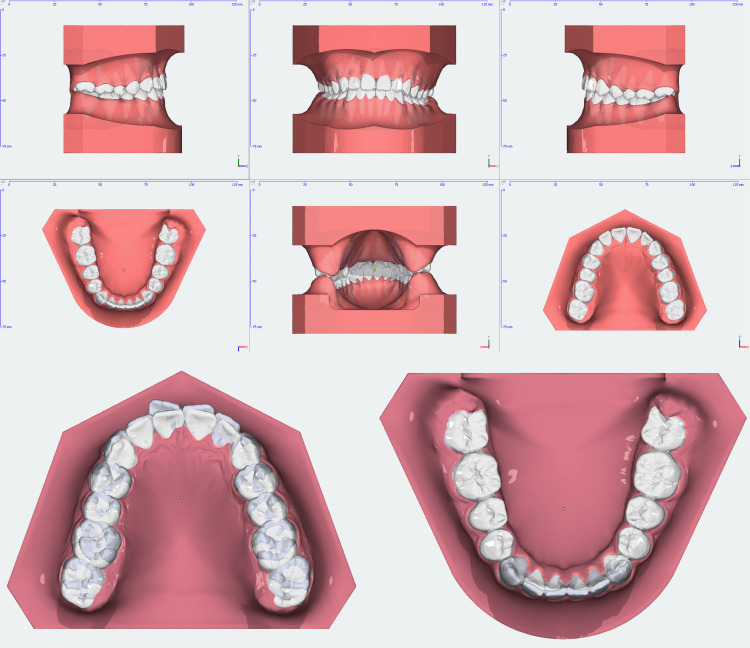
3D simulation of the treatment

**Figure 6:**
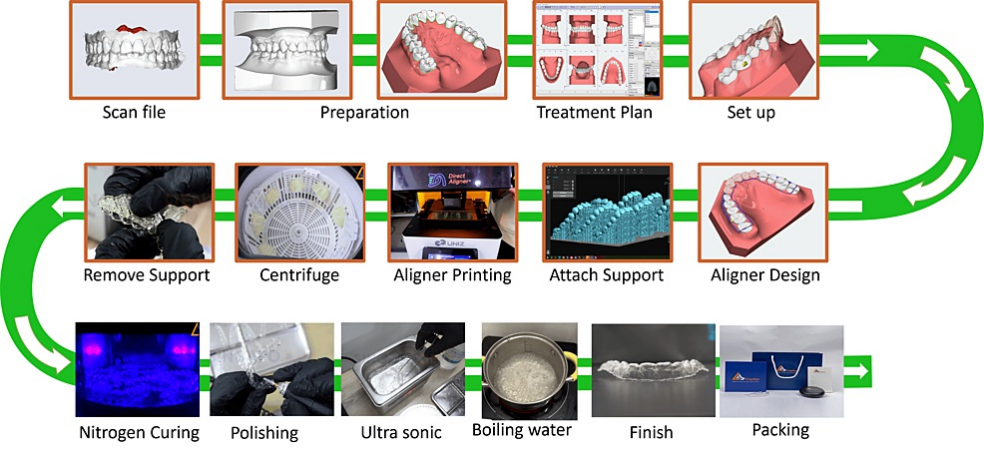
Laboratory procedure of the direct-printed clear aligner

**Figure 7:**
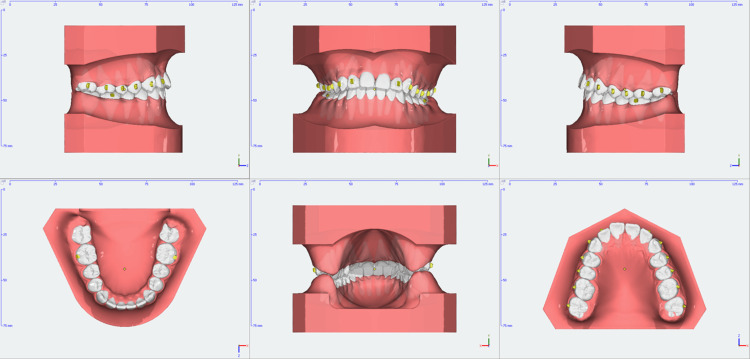
Position of the attachments and the first set of clear aligners

The initial step involved a 3D scan of the patient using an intraoral scanner and then a 3D simulation (Figure 5) before treatment using Onyxceph software (https://onyxceph.eu/en/) to check the biomechanics and movement of the treatment. We discussed with the parents and started to produce the direct printed aligners for the patient with the laboratory procedure, attachment design, and first setup for the treatment as shown in Figures 6, 7. After setting up the 3D digital design for treatment, we will begin the laboratory procedure by 3D printing the aligners. Next, we used a centrifuge machine for six minutes to remove all the resin inside the aligner, followed by removing the supports of the aligner. After that, we performed 20-minute nitrogen curing at level 2, trimmed the support, cleaned the aligners for 2 minutes in an ultrasonic cleaner at 80 degrees, and finally, submerged the aligners in boiling water for 1 minute before packing them. During the printing process, we used a Uniz Nbee 3D printer (UNIZ Technology, San Diego, CA, US), Tera Harz (https://itgraphy.com/ENG/) nitrogen curing, Tera Harz centrifuge, and ultrasonic cleaner with TC85-DAC resin. Optimized attachments were designed for mesial-distal movement in Onyxceph software. With the first set of aligners, we wanted to have the space by sequence distalization before alignment and retract the upper anterior using class II elastics from 6s lower to 3s upper. The production time for direct printed aligners is around 1 hour and 30 minutes for six aligners. The patient was instructed to use direct-printed aligners. Before inserting the aligners into the mouth, it was recommended to immerse them in warm water. This process softens the aligners and activates their shape memory effect, making it easier to fit them comfortably and reducing stress on the aligners. Additionally, the patient was advised to soak the aligners in boiled water every night to clean them thoroughly and prevent bacterial buildup.

**Figure 8:**
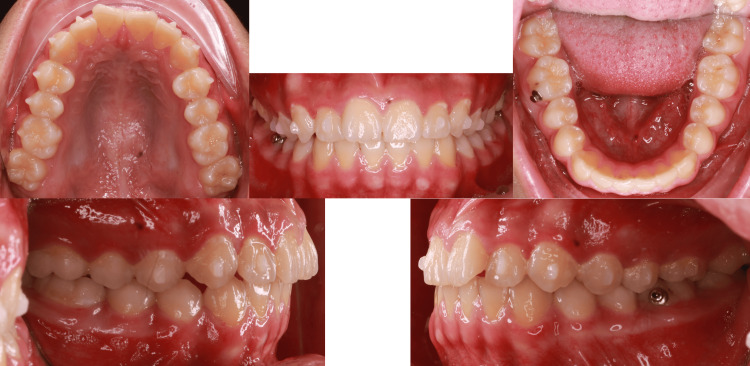
Treatment progress at six months

**Figure 9:**
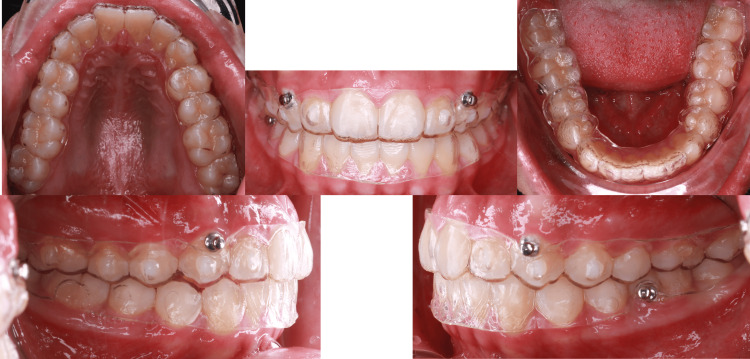
Direct-printed aligner in the mouth during the treatment

**Figure 10:**
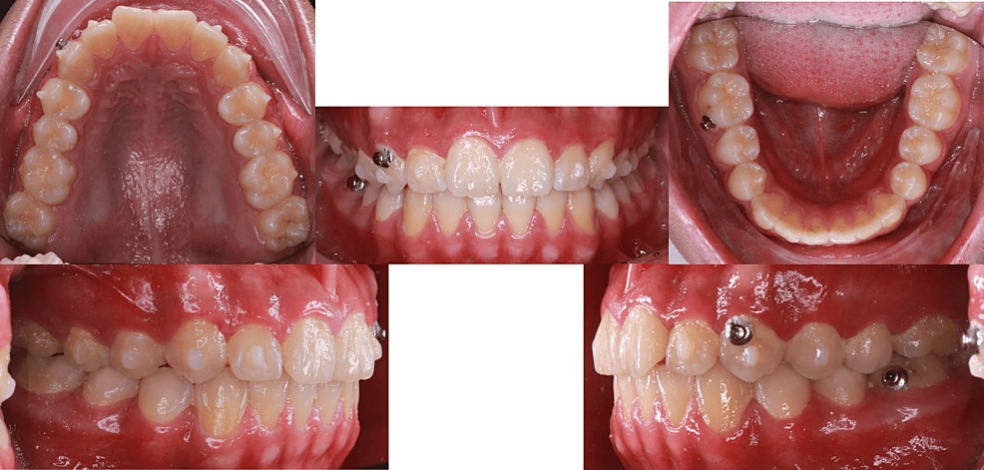
Treatment progress at nine months

**Figure 11:**
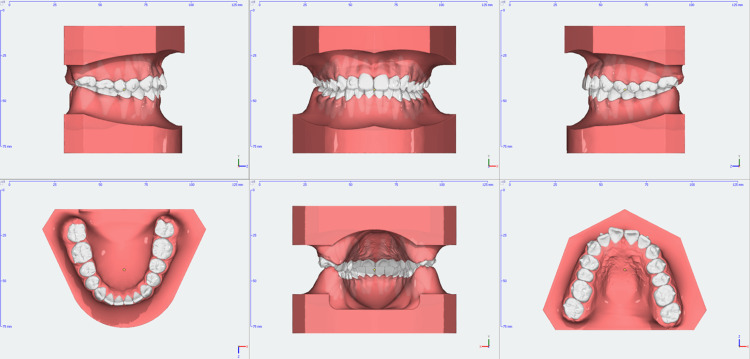
Digital model treatment progress at nine months

**Figure 12:**
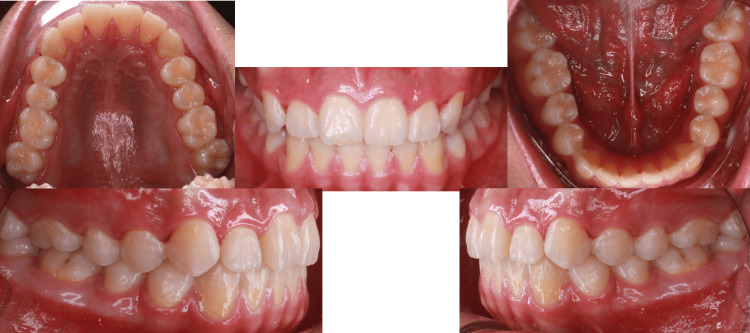
Intraoral pictures post-treatment

**Figure 13:**
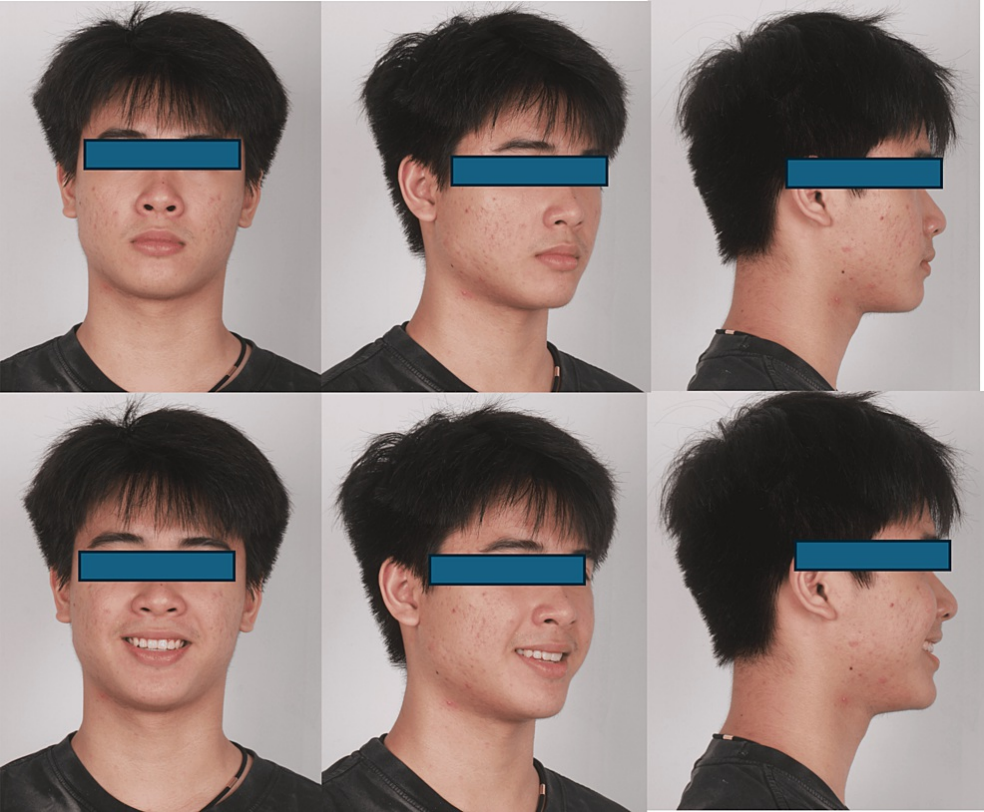
Extraoral pictures post-treatment

**Figure 14:**
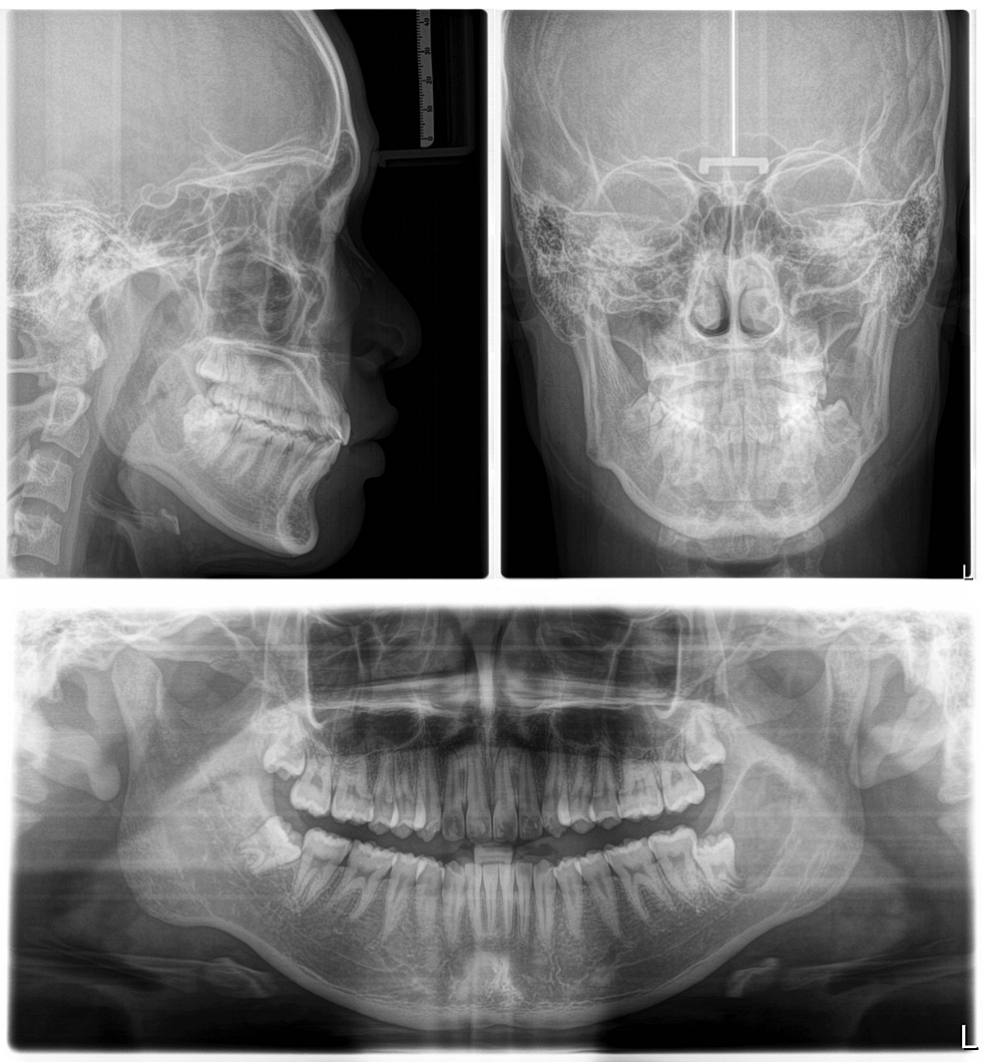
X-rays post-treatment

**Figure 15:**
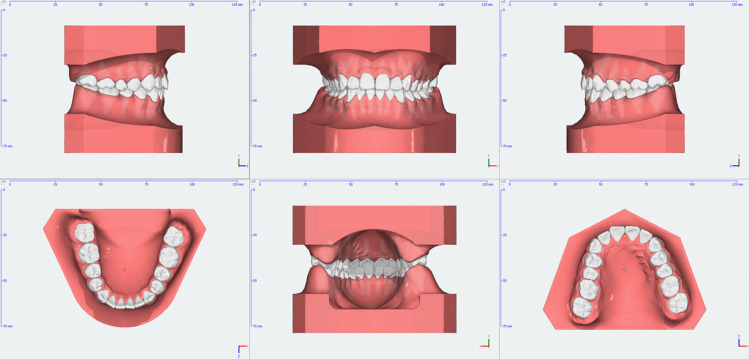
3D digital model post-treatment

During the treatment, we need to do refinements for the patient because the aligner can’t move the teeth 100%, as the digital setup, especially on tooth 11, but with a direct-printed aligner, the movement of the teeth is better than conventional thermoforming aligner with better control [8], the result after six months of the patient and direct printed aligner inside the mouth was showed in Figures 8, 9. After nine months, the treatment was almost finished, we just did some finishing to rotate the 11 (Figures 10, 11) perfectly. The treatment was finished after 12 months with 50 aligners on the upper and 20 aligners on the lower (Figures 12-15).

Treatment results

**Figure 16:**
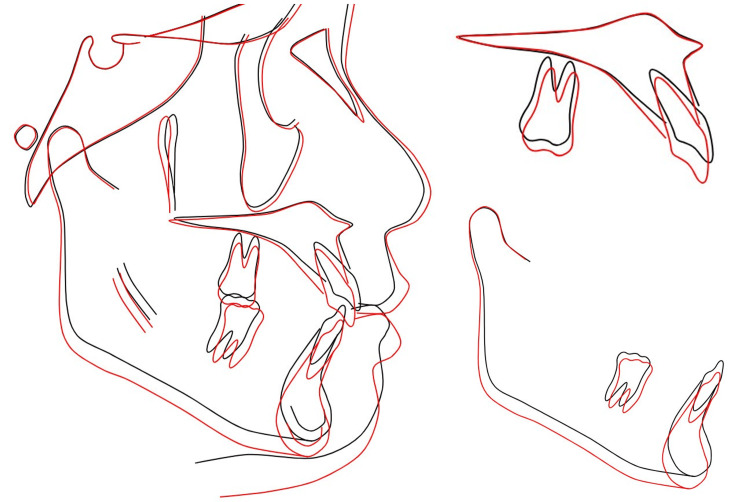
Cephalometric superimposition before and after treatment Black line: before treatment, red line: post-treatment

All treatment objectives were successfully achieved, resulting in well-aligned dentition and improved facial esthetics. The achieved outcomes include the maintenance of a Class I molar relationship and achieving the Class I canine relationship with better intercuspation. Cephalometric analysis and superimposition after treatment confirmed these changes, showing the improvement of skeletal relationship from Class II to Class I and a decrease of 8° in the inclination of the maxillary incisors. The limitation of the treatment outcome is the alignment was not perfect on UL2m U7s and LR2 but it was an acceptable treatment outcome with good occlusion and the patient was happy with the result (Figure 16 and Table 1).

**Table 1:** Cephalometric analysis pre-treatment and post-treatment ANB: A point, nasion, B point, FMA: Frankfort mandibular plane angle, IMPA: Incisor mandibular plane angle, L1: Lower central incisor, LL: Lower lip, MP: Mandibular plane, NA: Nasion point A NB: Nasion point B, SNA: Sella nasion point A, SNB: Sella nasion point B, U1: Upper central incisor, UL: Upper lip. E-line: Ricketts

Measurement	Norm	Pre-treatment	Post-treatment	
SNA (°)	81.1±3.7	87.1	85.4	SKELETAL
SNB (°)	79.2±3.8	82.8	82.8	
ANB (°)	2.5±1.8	4.3	3.6	
FMA (°)	25.0±4.0	23.4	22.2	
U1 – SN (°)	105.3±6.6	117.3	109.1	DENTAL
U1 - NA (mm)	4.0±3.0	4.7	3.6	
U1 - NA (°)	22.0±5.0	30.2	22.6	
U1 - L1 (°)	128.0±5.3	116.8	125.1	
L1 – NB (mm)	4.0±2.0	6.4	5.9	
L1 - NB (°)	25.0±5.0	28.7	28.7	
IMPA (°)	90.0±3.5	96.2	97.1	
UL – E line (mm)	0±2	-3	0.3	SOFT TISSUE
LL – E line (mm)	0±2	-2.1	1.4	

## Discussion

The use of clear aligner treatment began in 1945 and has evolved significantly over the past 80 years. Traditionally, dentists would order aligners from large companies or laboratories. However, recent advancements have allowed dentists to perform clear aligner treatments in their offices. Tozlu M et al. showed in-house aligners offer advantages such as lower costs, better control over delivery time, and easier treatment management [9]. In the past, clear aligners were typically produced using a thermoforming heating device, but the mechanical properties of thermoformed materials were not ideal. New technology now enables the direct printing of aligners, providing benefits such as faster procedures, improved fitting accuracy, and uniform thickness [10-12]. In 2022, Haris Pratsinis et al. showed that 3D direct-printed aligners are not cytotoxic for human gingival fibroblasts and do not have estrogenic effects [13]. Additionally, Jung-Yul Cha et al. have demonstrated that direct-printed aligners exhibit stable mechanical properties and are more resistant to deformation compared to thermoformed aligners [14]. In 2023, Kim et al. did some in-vitro research and concluded that directed printed aligners got lower, more consistent forces with fewer side effects and greater trueness and precision of direct-printed aligners than thermoformed aligners [15,16]. These advancements in clear aligner technology offer promising potential for improving orthodontic treatments.

Mild Class II malocclusion and crowding are common orthodontic issues that can be effectively treated using clear aligners. In-house direct-printed clear aligners refer to aligners manufactured using 3D printing technology. This allows for greater customization and precision in aligner fabrication, providing numerous advantages:

Effectiveness: Studies have shown that clear aligners can effectively correct mild to moderate Class II malocclusions through controlled tooth movement [17,18]. Advances in software technology allow for precise staging of tooth movements, enabling clinicians to plan and execute Class II correction efficiently. With a clear aligner, we can choose the teeth we want to move, and especially in house aligners, we can control the mechanics by ourselves. In some cases, the time for clear aligner treatment will be shorter than conventional braces.

Mechanics: Mechanically, Class II correction with clear aligners often involves the use of features like precision cuts, attachments, and elastics. Especially for mild Class II malocclusion, clear aligner treatment can be an efficient option for the patient with aesthetic and efficient treatment. These elements facilitate specific movements required to achieve proper occlusion and alignment. With an in-house clear aligner, we can control and change the mechanics during the treatment easily.

Comparative studies: Comparative studies between clear aligners and traditional fixed appliances for Class II correction have shown comparable outcomes in terms of treatment duration and effectiveness, with clear aligners offering advantages in terms of aesthetics and comfort.

Production time: The clinician can quickly deliver the aligners to the patient, possibly on the same day. It's a big advantage of in-house clear aligner treatment [19].

Cost of the treatment: Clinicians can produce aligners at a very economical cost compared to those bought from companies. The cost of a single aligner includes the cost of a 3D model print and plastic foil. Additionally, with the increase in the amount of software available for aligner fabrication, prices are expected to decrease rapidly. So when we want multidisciplinary treatment or orthodontic treatment before the prosthodontic treatment, with the low cost of an in-house direct-printed aligner, we can use it to reduce the treatment fee of the patient [19]. The cost of one direct-printed aligner is higher than that of a thermoforming aligner, but the tooth movement can be double that of a thermoforming aligner. Thus, if we can control it, the cost will be cheaper. With direct printed aligners, we have a new protocol and don't need to print the model, so it's more environmentally friendly than thermoforming aligners. In the future, the authors hope we can recycle clear aligners [20].

## Conclusions

In conclusion, in-house aligner treatment has the potential to revolutionize the economics of many practices. In-house production of direct-printed aligners offers many benefits for orthodontists and their patients, including shape memory effect, light force, biocompatibility, better physical and mechanical properties, as well as improved accuracy, fit, and clinical feasibility, representing a promising advancement in orthodontic treatment. Class II correction and crowding treatment using direct-printed clear aligners represent a promising advancement in orthodontics, offering patients a more comfortable and aesthetically pleasing alternative to traditional braces. However, it is crucial to strictly control and follow laboratory procedures to prevent allergic reactions and cytotoxicity in patients.
